# Autonomy, Competence, Relatedness, and Beneficence: A Multicultural Comparison of the Four Pathways to Meaningful Work

**DOI:** 10.3389/fpsyg.2018.01157

**Published:** 2018-07-10

**Authors:** Frank Martela, Tapani J. J. Riekki

**Affiliations:** ^1^School of Business/Department of Industrial Engineering and Management, Aalto University, Helsinki, Finland; ^2^Filosofian Akatemia, Helsinki, Finland

**Keywords:** autonomy, basic psychological needs, beneficence, cross-cultural, employee well-being, meaningful work, meaningfulness at work, work motivation

## Abstract

Meaningful work is a key element of positive functioning of employees, but what makes work meaningful? Based on research on self-determination theory, basic psychological needs, and prosocial impact, we suggest that there are four psychological satisfactions that substantially influence work meaningfulness across cultures: autonomy (sense of volition), competence (sense of efficacy), relatedness (sense of caring relationships), and beneficence (sense of making a positive contribution). We test the relationships between these satisfactions and perceived meaningful work in Finland (*n* = 594, employees of several organizations), India (*n* = 342, collected through Mturk), and the United States (*n* = 373, collected through Mturk). Regression analyses show that – except for competence in United States – all four satisfactions are significantly and independently associated with meaningful work. Moreover, structural equation modeling shows that they fully mediated the relationship between occupational position and work meaningfulness in India and in the United States. In sum, the results support the importance of these four satisfactions in explaining the psychological underpinnings of meaningful work.

## Introduction

Being able to experience meaningfulness is a fundamental part of having a life worth living (Camus, 1955; Wolf, 1997; Martela, 2017). Accordingly, a lack of meaning is associated with depression, mortality, and even suicide ideation (Harlow et al., 1986; Steger et al., 2006; Tanno et al., 2009). Given the decline in traditional sources of meaning such as religion, work has arguably become, along with family, one of the most important domains from which people derive meaningfulness in their lives (Baum and Stewart, 1990; Baumeister, 1991; Steger and Dik, 2009). This means that many employees around the world seek to find meaningfulness from their work. For example, a representative survey of the United States workforce show that 28% of respondents value purpose over money or status (Imperative, 2015), and another survey of 26,000 LinkedIn members in 40 countries show that 37% of respondents value purpose over money or status globally, with the number ranging from 53% in Sweden to 23% in Saudi Arabia (Hurst et al., 2016). Another probability sample of 1,200 college-graduated United States workers found that 34% of respondents are willing to accept 15% lower salary if that allows them to work for an organization whose values align with their own (Net Impact, 2012).

Finding one’s work meaningful and experiencing it as a calling have increasingly also become the focus of vocational and counseling psychology (Dik et al., 2009; Allan et al., 2017) in the quest to enhance the well-being and positive functioning of employees (Super, 1955; Robitschek and Woodson, 2006). Meaningfulness of work is closely linked to occupational callings (Duffy et al., 2012, 2013; Hirschi, 2012), and dignity at work (Di Fabio and Maree, 2016). In the last decades it has started to gain increased attention as an important psychological state on its own (Wrzesniewski, 2003; Michaelson, 2005; Rosso et al., 2010; Martela and Pessi, 2018). This research has demonstrated how meaningful work contributes to positive affective well-being (Arnold et al., 2007), occupational identification (Bunderson and Thompson, 2009), deriving benefits from a stressful work-related event (Britt et al., 2001), life satisfaction, less depression (Steger et al., 2012; Duffy et al., 2013), and finding one’s life meaningful (Steger and Dik, 2009). Accordingly, researchers have started to recognize that finding meaning in work is fundamental for work-related motivation, commitment, and overall well-being (Rosso et al., 2010) as well as career choices (Bunderson and Thompson, 2009; Dik et al., 2009). Accordingly, several scholars have recognized that meaningful work “should arguably be one of the most important questions for organizational scholarship” (Podolny et al., 2005, p. 1; see also Lepisto and Pratt, 2017). While previous work links meaningful work to important organizational outcomes such as less work absenteeism (Steger et al., 2012), decreased turnover intentions (Scroggins, 2008; Arnoux-Nicolas et al., 2016), and supervisor-rated performance (Harris et al., 2007), work meaningfulness is also valuable in its own right (Bowie, 1998; Roessler, 2012; Michaelson et al., 2014). Thus, striving for meaningful work is not just about obtaining certain outcomes, but being able to experience meaningfulness in one’s work activities is “an important humanistic endeavor in and of itself” (Lepisto and Pratt, 2017, p. 100), and part of what makes life good for human beings (Yeoman, 2014).

We define meaningfulness of work as the subjective experience of how significant and intrinsically valuable people find their work to be (Hackman and Oldham, 1975; Rosso et al., 2010). It is thus an overall evaluation of work as regards whether it is intrinsically valuable and worth doing. Thus, unlike work engagement (Bakker, 2011; Christian et al., 2011), psychological empowerment (Spreitzer, 1995), and job involvement (Brown, 1996), it is not primarily a motivational construct, but an evaluative construct. Thus, while finding one’s work meaningful often might lead to increased motivation, it is primarily about how much value we are able to derive from our work. It is also different from constructs such as job satisfaction, which is about evaluating one’s job with some degree of favor (Wright and Cropanzano, 2000). While we might draw satisfaction from many aspects of our job, like whether it is pleasurable, meaningfulness of work is more specifically about whether the work provides something that is in accordance with one’s values.

There is no lack of theoretical perspectives on meaningful work, but too few of these theoretical insights have been empirically tested. For example, Rosso et al. (2010, pp. 115, 119) call for more empirical research examining several potential sources of meaningful work simultaneously. Research on self-determination theory has argued that the psychological needs for autonomy, competence, and relatedness can be key predictors of meaning in life (Weinstein et al., 2012), and empirical research has shown this to be the case (Martela et al., 2017). We thus propose that these three needs are also likely to be key predictors of meaningful work. Furthermore, based on research showing the importance of beneficence and prosocial impact for meaningfulness (Van Tongeren et al., 2016; Allan et al., 2017; Martela et al., 2017), we suggest that it can be a fourth key factor associated with meaningful work. Furthermore, given that both the three psychological needs (e.g., Chen et al., 2015) and prosociality (e.g., Aknin et al., 2013) have been shown to be predictors of well-being cross-culturally, we propose that their relation to meaningful work should hold, disregarding the cultural context. Accordingly, using samples from three different continents and integrating research on meaningful work with research on meaning in life, in this paper we test empirically how autonomy, competence, relatedness, and beneficence – that have been recognized within psychology as important sources of wellness and meaningfulness – are associated with finding work meaningful.

## Autonomy, Competence, and Relatedness as Predictors of Meaningful Work

Following previous work (Baumeister, 1991; Steger and Dik, 2009; Allan et al., 2015) we see that meaningfulness as regards life and meaningfulness as regards work are facets of the same psychological construct. As regards potential sources of meaningfulness, self-determination theory has become one of the most cited contemporary theories of human motivation and wellness (Ryan and Deci, 2000, 2017). Research within self-determination theory has identified three basic psychological needs: autonomy, competence, and relatedness (Ryan and Deci, 2000, 2017) that have been shown to play an important role for the motivation, well-being, life satisfaction, and vitality of people on both general and daily level (e.g., Reis et al., 2000; Ryan et al., 2010; Martela and Ryan, 2016b). Self-determination theory has been increasingly applied to the work context as well and several studies (reviewed in Van den Broeck et al., 2016; Deci et al., 2017), show that these needs explain, for example, vigor and less exhaustion at work (Van den Broeck et al., 2010). *Autonomy* refers to a sense of volition and internal perceived locus of causality in one’s undertakings. The person feels that the actions emanate from the self and reflect who one really is, instead of being the result of external pressures. *Competence*, in turn, is about a sense of mastery and efficacy in one’s activities. One feels that one is capable at what one does and is able to accomplish projects and achieve one’s goals. *Relatedness* is more about the interpersonal dimension, reflecting the extent to which a person feels that one is connected to others, has caring relationships, and belongs to a community. The theory argues that these three needs are universal in the sense that their relationship to wellness and positive functioning should remain robust no matter the cultural context (Deci and Ryan, 2000; Ryan and Deci, 2017), and empirical research demonstrates that the three needs are associated with well-being in countries ranging from United States, Australia, and Belgium to Mexico, Peru, Malaysia, China, and Philippines (Church et al., 2013; Chen et al., 2015).

In addition to the role of these three needs as robust predictors of well-being and positive human functioning, Weinstein et al. (2012) recently argued that the three needs for autonomy, competence, and relatedness should be seen as serious candidates for what makes life meaningful. When we are autonomously able to choose our activities and work tasks, these are prone to be experienced as more meaningful. Empirical work has supported this suggestion (Ryff, 1989; McGregor and Little, 1998). Such autonomy and self-determination is also closely linked with psychological empowerment at work (Spreitzer, 1995; Seibert et al., 2011). Similarly, if we feel ineffective, incompetent, and unable to accomplish our tasks, this makes our efforts feel meaningless (Baumeister, 1991; Steger et al., 2008), while feeling of competence leads to psychological empowerment (Spreitzer, 1995; Seibert et al., 2011). Finally, when people are asked what makes their lives meaningful, relationships with family and friends are mentioned as a key source of meaning by the majority of people (Lambert et al., 2010). The evidence about the causal impact of relatedness on meaning in life is quite robust (Stillman et al., 2009; Lambert et al., 2013). Accordingly, the three psychological needs all seem to play a role in explaining what makes life meaningful. Recent work indeed shows that all three needs are independently connected to meaning in life, even after controlling for the influence of each other (Martela et al., 2017). Given the close connection between meaningfulness as regards life and meaningfulness as regards work (Steger and Dik, 2009; Allan et al., 2015), we expect these three needs to have positive associations also with meaningful work. Furthermore, given the cross-cultural predictive power of the three needs, we expect this to be true no matter the cultural context. However, previous empirical work has not examined the relationship between these three needs and meaningful work. Thus, the first hypothesis we want to examine in this study is as follows:

*Hypothesis 1*: Autonomy, competence, and relatedness all have independent relations with meaningful work, even when controlling for the influence of the other needs, and these relations will hold no matter the cultural context.

## Beneficence As A Predictor of Meaningful Work

Increasing amount of research has also demonstrated that prosocial behavior – doing something to benefit other people – is beneficial for one’s own well-being (e.g., Dunn et al., 2008; Martela and Ryan, 2016a). Furthermore, these research findings have been replicated in countries around the world from Canada to India and from South Africa (Aknin et al., 2013) to a small-scale rural society on the Pacific Island of Vanuatu (Aknin et al., 2015). Based on these results, the researchers suggest that emotional benefits derived from prosocial spending is a “psychological universal” (Aknin et al., 2013). Other work has investigated whether it could be a psychological need on par with the three established needs for autonomy, competence, and relatedness (Martela and Ryan, 2016b).

At the same time, many theorists have emphasized how beneficence – the sense of having a prosocial impact – can be a key source of meaningfulness (Frankl, 1963; Wong, 1989). Various forms of benevolent behavior or attitudes have been empirically connected to a greater sense of meaning (Shek et al., 1994; Schnell, 2011). For example, Van Tongeren et al. (2016) showed in four studies both cross-sectional and experimental evidence for the important role of prosocial behavior in evaluations of meaning in life. Most importantly for our present investigation, Allan et al. (2017) demonstrated in three experimental studies that helping others increases people’s sense of task and work meaningfulness (see also Colbert et al., 2016). Therefore, having a prosocial impact can be a key source of meaningful work. However, to establish that the connection between having a prosocial impact and meaningful work is not explained by some other factor – e.g., increased sense of competence from having a prosocial impact – it would be important to control for the influence of autonomy, competence, and relatedness. A recent study demonstrated that prosocial impact had an independendent relation with meaning in life, even when controlling for the influence of autonomy, competence, and relatedness (Martela et al., 2017). But no study has looked whether there would be a relation between prosocial impact and meaningful work when controlling for autonomy, competence, and relatedness. Given the research demonstrating that the positive wellness effects of prosocial impact is likely to be a ‘psychological universal’ (Aknin et al., 2013, 2015), we also expect the connection between beneficence and meaningful work to remain robust cross-culturally. This leads to our second hypothesis:

*Hypothesis 2:* Beneficence has an independent relation with meaningful work, even when controlling for the influence of the three psychological needs for autonomy, competence, and relatedness, and this relation will hold no matter the cultural context.

## Occupational Position and Meaningful Work

Finally, it has been suggested that having a lower position within the organizational hierarchy could diminish one’s ability to experience work as meaningful (Bowie, 1998). At the same time, autonomy, competence, and relatedness have been shown to be positively correlated with occupational position (González et al., 2016), and with meaning in life (Martela et al., 2017). Previous research has shown that type of motivation can mediate the relationship between social class and meaningful work (Allan et al., 2016), while autonomy, competence, and relatedness have been shown to mediate the relation between socioeconomic status and physical and mental health (González et al., 2016). The psychological satisfactions thus seem to have the capacity to act as mediators between socioeconomic position and well-being, as suggested by self-determination theory (Ryan and Deci, 2017). Accordingly, Allan et al. (2016) call for research that would look at the capacity of basic psychological needs to mediate the relation between various aspects of social class and meaningful work. In line with this, we suggest that being in a higher occupational position can give the employee more possibilities to make volitional choices as regards one’s work (autonomy), to experience more mastery and sense of accomplishment as regards one’s work tasks (competence), to increase the quality of one’s interactions by having more choice over with whom to interact during the workday (relatedness), and to experience that the positive impact of one’s work is broader (beneficence). Therefore, the final hypothesis we want to test is the following:

*Hypothesis 3: S*ense of autonomy, competence, relatedness, and beneficence mediate the expected positive connection between having a higher occupational position and having a more meaningful work.

All in all, the research study thus answers calls for more empirical research on the key sources on meaningful work, especially research that would examine more than one source simultaneously, and research that would examine the topic cross-culturally.

## Study 1

In the first study, we wanted to test the hypothesis that the three psychological needs and beneficence, operationalized as a sense of prosocial impact, all have an independent relation to meaningful work when controlling for each other. We wanted to see how much they could together explain of the variance in people’s evaluations of meaningfulness of work.

### Participants and Procedure

This study was carried out in accordance with the recommendations of the Ethical Review Board of the University of Helsinki. In accordance with the Declaration of Helsinki, we sought informed consent from all study participants, and they gave their consent anonymously in the online form. We used a combined sample collected from three separate sources. The first participant group were employees of a Finnish subsidiary of a multinational technology company (*n* = 334), and the second group consisted of employees of a Finnish subsidiary of a European plastic product manufacturer company (*n* = 85). These participants filled the survey anonymously as part of a volunteer internal workplace audit. The third group of participants consisted of employees of several different organizations who took part of a leadership training program and were mostly HR-specialist and middle-level/higher-level management (*n* = 175). They filled the questionnaire voluntarily as part of the training program. In total 667 employees in these three groups answered the survey, and 89% of the participants gave informed consent to use their answers for scientific research leading to a total sample size of 594.

We administered the survey in Finnish language with the questions translated from English and then back-translated by a professional translator to ensure accuracy of translation, with 56% of participants in the age group 41–60, 39% in the age group 26–40, 2% under 26, and 3% over 60. Positions in the organizations were: 9.9% manufactory workers, 61% specialists/employees, 19% middle management, and 9.3% higher management role.

### Measures

#### Work Meaningfulness

To assess work meaningfulness, we administrated three face valid questions: ‘I feel that my work has a meaning,’ ‘My work gives me a feeling of meaningfulness,’ and ‘Our work community does something valuable.’ The participants were asked to rate the extent to which they agreed with these statements on a scale from 1 (do not agree) to 7 (fully agree) (α = 0.91).

#### Basic Psychological Satisfactions

For satisfaction of SDT’s three basic needs for autonomy, competence, and relatedness, we used the satisfaction items from the work adjusted version (Schultz et al., 2014) of the Basic Need Satisfaction and Frustration Scales (Chen et al., 2015). The scale includes four items measuring satisfaction of each of the three needs, e.g., “I feel my choices on my job express who I really am” for autonomy (α = 0.75), “At work, I feel capable at what I do” for competence (α = 0.83), and “I feel connected with people who care for me at work, and for whom I care at work” for relatedness (α = 0.88). For sense of beneficence, we used a work adjusted version of the Beneficence Scale developed by Martela and Ryan (2016b). It included four items, e.g., “I feel that my actions at work have a positive impact on the people around me (α = 0.89).” Items for all four satisfactions were mixed together and rated on a scale from 1 (do not agree) to 7 (fully agree).

### Results and Discussion

#### Preliminary Analysis

**Table 1** presents the means, standard deviations, and intercorrelations of work meaningfulness and the four psychological satisfactions. As expected, and analogously to what has been found in research on psychological needs in life in general (Martela and Ryan, 2016b; Martela et al., 2017), the three needs for autonomy, competence, and relatedness were correlated positively with each other, with beneficence, and all four factors were also positively correlated with meaningful work.

**Table 1:** Study 1 means, standard deviations, and zero-order correlations between study variables.

	*M*	*SD*	1	2	3	4
1. Meaningful work	5.27	1.35	–			
2. Autonomy	4.64	1.15	0.622	–		
3. Competence	5.53	0.93	0.531	0.438	–	
4. Relatedness	5.38	1.06	0.496	0.439	0.438	–
5. Beneficence	4.74	1.34	0.730	0.669	0.504	0.444

*All correlations significant on a 99% confidence level.*

To test whether the four satisfactions form independent factors, we performed a confirmatory factor analysis with maximum likelihood and four separate factors, using lavaan package in RStudio 1.0. Recognizing that the cutoff values should not be seen as more than rough rules-of-thumb (Hu and Bentler, 1999; Marsh et al., 2004), we adopted the following cutoff values for adequate fit based on previous recommendations (Browne and Cudeck, 1993; Hu and Bentler, 1999; Marsh et al., 2004): CFI, TLI > 0.90, RMSEA, SRMR < 0.08. The fit indices of the model [χ^2^ (df = 98) = 454.998, CFI = 0.931, TLI = 0.916, RMSEA = 0.078, SRMR = 0.053] were within these standards for acceptable fit^1^, and each of the items also loaded significantly on the intended latent factor. More importantly, as cut off points are always more or less arbitrary (Marsh et al., 2004), we wanted to compare the model fit with two alternative models. First, a model where all the items for all four satisfactions were taken as indicators of the same underlying construct [χ^2^ (df = 104) = 1843.303, CFI = 0.665, TLI = 0.613, RMSEA = 0.168, SRMR = 0.111]. Second, as previous research has sometimes treated the three psychological needs of SDT as one aggregate factor (e.g., Deci et al., 2001; Van den Broeck et al., 2008), we tested a model where the items for the three needs of SDT were taken as indicators of one factor, and beneficence items formed a separate factor [χ^2^ (df = 103) = 1433.904, CFI = 0.744, TLI = 0.701, RMSEA = 0.147, SRMR = 0.103]. As can be seen, the hypothesized model of four separate factors compared favorably to the alternative models.

#### Primary Analysis

In line with previous research that has examined the individual contributions of each of the need satisfactions (e.g., Sheldon and Niemiec, 2006; Van den Broeck et al., 2010; Martela and Ryan, 2016b), we used a stepwise regression analysis with meaningful work as the dependent variable to look at whether all four satisfactions have a direct connection with meaningfulness after controlling for each other. In the first step we entered sample group, position and age as control variables *F*(3,589) = 5,014 *p* = 0.002, *R*^2^ = 0.03. While sample group (β = 0.007, *p* = 0.885) and position (β = 0.089, *p* = 0.084) did not have a significant relationship with meaningfulness, age had a significant relation (β = 0.118, *p* = 0.004) with older participants experiencing their work as more meaningful. In step 2, the three psychological needs were simultaneously entered as independent variables. In the regression analysis *F*(6,586) = 100, *p* < 0.001, *R*^2^ = 0.50 autonomy (β = 0.414, *p* > 0.001), competence (β = 0.254, *p* > 0.001), and relatedness (β = 0.208, *p* > 0.001) all had independent relations with meaningful work. In step 3, beneficence was added as an independent variable. The regression analysis *F*(7,585) = 131, *p* < 0.001, *R*^2^ = 0.61 showed that autonomy (β = 0.177, *p* > 0.001), competence (β = 0.146, *p* > 0.001), relatedness (β = 0.150, *p* > 0.001), and beneficence (β = 0.464, *p* > 0.001) all were significantly related to meaningful work. Also, age (β = 0.074, *p* = 0.005) remained a significant predictor of meaningful work. Furthermore, in both steps 2 and 3, the increase in variance explained was significant (*p* < 0.001). Given the relatively high correlations between the four satisfactions, we examined the variance inflation factors (VIFs) to see whether multicollinearity might be problematic for the model. The VIF statistics for all four predictors were between 1.3 and 2.1, indicating that multicollinearity was not a problem (to detect problems with multicollinearity, the most commonly used threshold is VIF > 10, while sometimes a more conservative VIF > 5 is used, see Cohen et al., 2013; Dormann et al., 2013).^2^

#### Interpretation

The results showed that both the three psychological needs and beneficence explained independent variance in meaningful work that cannot be reduced to the other independent variables. Together with the control variables, they explained 61% of the total variance in people’s experience of meaningful work (when control variables were excluded, the variance explained remained at 61%). The standardized estimates and the total variance explained are similar in size as has been found as regards the connection between the four satisfactions and meaning in life (Martela et al., 2017). Interestingly, although the organizations in the study were operating in different fields and people were working in various positions from manufactory workers to higher management, the same basic satisfactions seemed to be relevant in all organizations and positions.

## Study 2

To increase the cross-cultural generalizability of the findings of Study 1, we wanted to collect two new samples from two different continents to test the same hypotheses as in Study 1: Whether the three psychological needs and beneficence will simultaneously explain variance in meaningful work. Our aim was thus not to compare the cultures but rather to test the same hypotheses in three separate cultures, in order to get some support for the suggestion that the hypotheses would hold, no matter the cultural context. Additionally, we also tested the hypothesis that the four satisfactions mediate the relation between occupational position and meaningful work.

### Participants and Procedure

To increase the cross-cultural generalizability of the findings of Study 1, we collected two samples, one from United States and one from India. Both studies were carried out in accordance with the recommendations of the Ethical Review Board of the University of Helsinki. In accordance with the Declaration of Helsinki, we sought informed consent from all study participants, and they gave their consent anonymously in the online form. The English language survey items used in both populations were identical and administrated through Amazon Mechanical Turk (Mturk), which has in recent years become increasingly popular source of study participants in behavioral sciences (see Buhrmester et al., 2011; Mason and Suri, 2012). Several studies have been conducted to examine the demographics of the participants and although they are far from being representative of the whole population, it has been concluded that Mturk is a “reliable and cost-effective source of high-quality and representative data, for multiple research purposes” (Peer et al., 2017, p. 153). In both countries, our aim was to have at least 300 participants in the sample. A *post hoc* power analysis revealed that given alpha of 0.05 and desired power of 0.80, such sample size should be able to detect effect sizes above 0.04.

For India, 452 participants answered the whole survey, but we excluded 110 participants for either answering the survey faster than the *a priori* estimated cut off point of 2.45 min or for failing to answer correctly on the Instructional Manipulation Check (Oppenheimer et al., 2009) question (‘It’s important that you pay attention to the questions. Please click ‘strongly disagree’.’). Final sample size was thus 342. Using such measures to exclude inattentive participants is a relatively standard procedure when using Mturk samples and has been shown to improve the data quality and statistical power (e.g., Maniaci and Rogge, 2014). Nevertheless, to examine that our chosen cutoff point didn’t affect the results, we recalculated the primary analyses including all the excluded participants, and this did not affect the main results.

We excluded two other participants as they reported being currently unemployed, leaving a final sample size of 342. Of the participants, 73% reported being male and 27% female, the ethnicity of the participants was predominantly South Asian (88%) with 6% East Asian, and 6% identifying with other ethnicities. The age range was from 18 to 61 with the average age being 31. As regards the position within the organization, 3% reported being top level management, 33% middle level management, 20% first level management, 21% knowledge worker/specialist, 7% blue-collar worker/other, 5% first-line worker, 7% self-managed worker, and 5% trainee.

As regards the United States sample, 587 participants answered the whole survey. We excluded 203 participants using the same criteria as in the Indian sample. Additionally, 17 participants who reported being currently unemployed were excluded, leaving a final sample size of 373. Of the participants, 53% reported being male, 47% female, and 0.8% preferring not to say. The ethnicity of the participants was mainly Caucasian (75%) with 7% East Asian, 6% African American, 4% Hispanic, and 8% identifying with other ethnicities. The age range was from 18 to 66 with the average age being 37. As regards the position within the organization, 4% reported being top level management, 9% middle level management, 14% first level management, 35% knowledge worker/specialist, 12% blue-collar worker/other, 12% first-line worker, 14% self-managed worker, and 1% trainee.

### Measures

#### Work Meaningfulness

To assess work meaningfulness, we used the positive meaning subscale from the Work and Meaning Inventory (WAMI) (Steger et al., 2012). It included four items (e.g., I have a good sense of what makes my job meaningful) with an internal reliability of 0.83 in India, and 0.93 in United States. The participants were asked to indicate how true each statement was as regards their work, on a scale from 1 (Absolutely untrue) to 5 (Absolutely true).

#### Basic Psychological Satisfactions

For satisfaction of SDT’s three basic needs for autonomy, competence, and relatedness, and for beneficence satisfaction, we used the same four items per satisfaction as were used in Study 1. The reliabilities of the scales were α = 0.88 (India) and α = 0.90 (United States) for autonomy, α = 0.81 and α = 0.87 for competence, α = 0.83 and α = 0.94 for relatedness, and α = 0.83 and α = 0.92 for beneficence.

#### Control Variables

As control variables, we asked participants to indicate their age, gender, ethnicity and position in the organization.

### Results and Discussion

#### Preliminary Analysis

**Table 2** presents the means, standard deviations, and intercorrelations of work meaningfulness and the four psychological satisfactions in both samples. As in Study 1, all five study variables were positively correlated with each other. To test whether the four satisfactions form independent factors, we performed a confirmatory factor analysis similarly as in Study 1. The fit indices in both the United States sample [χ^2^ (df = 98) = 315.156, CFI = 0.956, TLI = 0.946, RMSEA = 0.077, SRMR = 0.051] and the Indian sample [χ^2^ (df = 98) = 221.372, CFI = 0.958, TLI = 0.948, RMSEA = 0.061, SRMR = 0.035] demonstrated adequate fit, using the same standards as in Study 1. And again, the four-factor model compared favorably in both samples with a model where the items for all four satisfactions are part of one overall factor [United States: χ^2^ (df = 104) = 1595.636, CFI = 0.699, TLI = 0.653, RMSEA = 0.196, SRMR = 0.106; India: χ^2^ (df = 104) = 326.603, CFI = 0.924, TLI = 0.912, RMSEA = 0.079, SRMR = 0.044], and with a model where the three needs of SDT form one factor and beneficence another factor [United States: χ^2^ (df = 103) = 1269.178, CFI = 0.765, TLI = 0.726, RMSEA = 0.174, SRMR = 0.104, India: χ^2^ (df = 103) = 289.147, CFI = 0.936, TLI = 0.925, RMSEA = 0.073, SRMR = 0.041].

**Table 2:** Study 2 means, standard deviations, and zero-order correlations between study variables.

	*M* (India/United States)	*SD* (India/United States)	1	2	3	4	5
1. Meaningful work	3.98/3.48	0.69/1.06	–	0.815	0.487	0.629	0.717
2. Autonomy	5.52/4.70	0.95/1.48	0.721	–	0.507	0.673	0.745
3. Competence	5.77/5.86	0.84/0.99	0.666	0.739	–	0.484	0.502
4. Relatedness	5.54/4.82	0.93/1.47	0.670	0.744	0.694	–	0.612
5. Beneficence	5.44/4.84	0.98/1.43	0.701	0.737	0.672	0.724	–

*Correlations for India below the diagonal, for United States above the diagonal. All correlations significant on a 99% confidence level.*

#### Primary Analysis

Following the approach taken by Church et al. (2013) in their study of the independent contributions of psychological needs on well-being in eight cultures, we conducted a stepwise regression analysis separately for both countries with meaningful work as the dependent variable, to examine the direct effects on meaningfulness of the four satisfactions when controlling for each other. As regards the Indian sample, in the first step we entered age, gender, ethnicity and occupational position as control variables *F*(4,337) = 6,89, *p* < 0.001, *R^2^* = 0.08. While ethnicity did not have a relationship with meaningfulness (*p* = 0.487), age (β = 0.152, *p* = 0.006), position (β = 0.134, *p* = 0.014) and gender (β = 0.148, *p* = 0.005) were significantly associated with meaningfulness of work, with older people, people higher up in the organization, and women experiencing their work as more meaningful. In step 2, the three psychological needs were simultaneously entered as independent variables. The regression analysis *F*(7,334) = 68.1, *p* < 0.001, *R^2^* = 0.59 showed that autonomy (β = 0.383, *p* > 0.001), competence (β = 0.222, *p* > 0.001), and relatedness (β = 0.219, *p* > 0.001) all had independent relations with meaningful work. In step 3, beneficence was added to the model as an independent variable. The regression analysis *F*(8,333) = 66.5, *p* < 0.001, *R^2^* = 0.62 showed that autonomy (β = 0.288, *p* > 0.001), competence (β = 0.175, *p* = 0.002), relatedness (β = 0.130, *p* = 0.024) and beneficence (β = 0.269, *p* > 0.001) were all significantly related to meaningful work. Interestingly, age (β = 0.020, *p* = 0.591) and occupational position (β = 0.008, *p* = 0.830) were no longer associated with meaningful work, when the four satisfactions were included in the model. The increase in variance explained in steps 2 and 3 was statistically significant (*p* < 0.001).

As regards the United States sample, we again entered age, gender, ethnicity and occupational position in the first step as control variables *F*(4,368) = 5.56, *p* < 0.001, *R*^2^ = 0.06. While gender (β = 0.011, *p* = 0.828) and ethnicity (β = 0.012, *p* = 0.818) did not have a relationship with meaningfulness, age (β = 0.174, *p* = 0.001) and position (β = 0.139, *p* = 0.007) were significantly associated with meaningfulness of work, with older people and people higher up in the organization experiencing their work as more meaningful. In step 2, the three psychological needs were simultaneously entered as independent variables. The regression analysis *F*(7,365) = 114.4, *p* < 0.001, *R^2^* = 0.69 showed that autonomy (β = 0.685, *p* > 0.001) and relatedness (β = 0.132, *p* = 0.001) were significantly related to meaningful work, but competence (β = 0.056, *p* = 0.118) wasn’t. In step 3, beneficence was added as an independent variable. The regression analysis *F*(8,364) = 109.3, *p* < 0.001, *R^2^* = 0.71 showed that autonomy (β = 0.562, *p* > 0.001), relatedness (β = 0.096, *p* = 0.019), and beneficence (β = 0.217, *p* > 0.001) were all significantly related to meaningful work. However, competence (β = 0.027, *p* = 0.444) was not related to meaningful work. Interestingly, while age (β = 0.069, *p* = 0.024) remained a significant predictor, occupational position (β = 0.045, *p* = 0.119) was no longer associated with meaningful work, when the four satisfactions were included in the model. The increase in variance explained in steps 2 and 3 was statistically significant (*p* < 0.001). Last, we tested for multicollinearity by examining the VIF’s. In both the United States sample (VIF’s between 1.5 and 2.8) and in the Indian (VIF’s between 2.5 and 3.3) multicollinearity seemed to not be a problem.

#### Mediation Analysis

To test the hypothesis that the four psychological satisfactions mediate the relation between occupational position and meaningful work, we decided to use structural equation modeling (SEM) that can take into account the observed correlations between the four satisfactions. Accordingly, we specified a model where occupational position (X) had a direct path to meaningful work (Y), as well as indirect paths through each of the four satisfactions (M_1_, M_2_, M_3_, M_4_). The indirect effects were defined as the product of the two paths linking X to Y via the mediator. Furthermore, the four satisfactions were set to correlate with each other. As regards the Indian sample, the model fit was good [χ^2^ (df = 175) = 360.564, CFI = 0.951, TLI = 0.941, RMSEA = 0.056, SRMR = 0.036]. Examination of the direct and indirect effects showed that while the direct effect was non-significant (β = -0.011, *p* = 0.608) the total indirect effect was significant (β = 0.106, *p* = 0.001), indicating full mediation. Of the individual indirect effects, however, only the one through autonomy (β = 0.072, *p* = 0.063) was marginally significant. Thus, while mediation didn’t seem to be strongly related to any of the individual satisfactions, together they fully mediated the relationship between occupational position and meaningful work.

**FIGURE 1:**
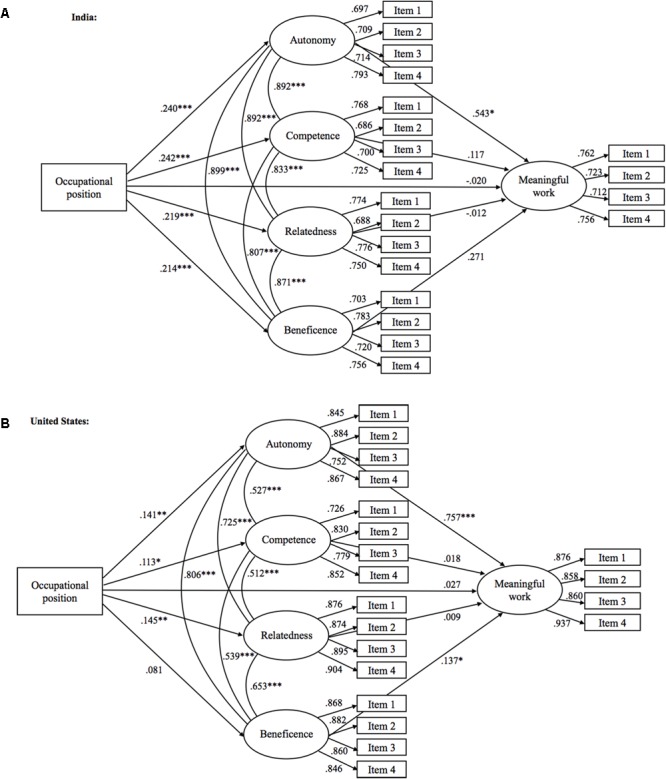
The mediation models showing the standardized paths between study variables in India **(A)** and in United States **(B)**. Error terms omitted for clarity. ^∗^*p* < 0.05, ^∗∗^*p* < 0.01, ^∗∗∗^*p* < 0.001.

As regards the United States sample, the model fit was acceptable [χ^2^ (df = 175) = 545.860, CFI = 0.946, TLI = 0.935, RMSEA = 0.075, SRMR = 0.050]. Examining the direct and indirect effects again showed that while the direct effect was non-significant (β = 0.027, *p* = 0.137) the total indirect effect was significant (β = 0.101, *p* = 0.002), indicating full mediation. Of the individual indirect effects, only the one through autonomy, (β = 0.065, *p* = 0.011) was significant. Thus it seemed that the relation between occupational position and meaningful work was mostly mediated by the higher degree of autonomy people higher up in the organizational hierarchy experienced (**Figure 1**).

#### Interpretation

These results mainly replicated the results from Finland in two samples from United States and India. In India, all four satisfactions emerged as independently predicting variance in meaningful work, while in United States all but competence emerged as independent predictors. Together with the control variables these four satisfactions were able to explain 62% of variance in meaningful work in India and 71% in United States (61% and 70%, when control variables were excluded from the analysis). We controlled for age, gender, ethnicity, and position within the organization, thus showing that these factors cannot explain the connection between the four satisfactions and meaningful work. In fact, the results in both India and United States showed that the four satisfactions fully mediated the significant relations between occupational position and meaningful work, with autonomy emerging as the key mediator. This suggests that the reason why people higher up in the organizational hierarchy in both India and United States experienced their work as more meaningful might have been the fact that they experienced more satisfaction on these four psychological factors, especially autonomy. This lends further support for the importance of these four factors in explaining what makes work meaningful.

## Discussion

The present results provide one of the very first cross-cultural tests of potential psychological underpinnings of meaningful work. In three different countries on three different continents, we tested whether the satisfaction of autonomy, competence, relatedness, and beneficence would all have independent predictive value in explaining how meaningful people evaluate their work to be. In Finland and India all four satisfactions turned out to be independent and significant predictors of meaningful work. In United States, the relation between competence and meaningful work was rendered non-significant when controlling for the other three satisfactions, but the remaining three satisfactions were all significantly and independently associated with meaningful work. These results remained significant, when controlling for age, occupational position, gender and ethnicity. In other words, at least for autonomy, relatedness and beneficence, we find consistent cross-cultural support for their role in evaluations of meaningful work. As regards competence, its connection to meaningful work evaluations was supported in two out of three countries studied. Looking beyond mere statistical significance, it was shown that the four satisfactions taken together were able to explain 60% to 70% of the total variance in meaningful work evaluations. This means that if we know how satisfied people are as regards autonomy, competence, relatedness, and beneficence in their work, we already can predict with relatively good accuracy how much meaningfulness they experience in their work.

Furthermore, we also examined whether the four satisfactions can explain the observed relationship between occupational position and meaningful work in the United States and India samples. The results of SEM demonstrated that the four satisfactions fully accounted for this relationship in both countries with autonomy playing a key role in mediating the connection between occupational position and meaningful work. These results thus suggest that the reason people higher up in the organizational hierarchy experience more meaningfulness at work could be related to the fact that people higher up in organization typically have more autonomy as regards their work.

### Similarities and Differences With Previously Suggested Predictors of Meaningful Work

Within research on meaningful work, Marjolein Lips-Wiersma identified through a qualitative psychobiographical study (Lips-Wiersma, 2002) four central content dimensions that she later argued “make up MFW [meaningful work] itself” (Lips-Wiersma and Wright, 2012, p. 659). These dimensions are (1) developing and becoming self, (2) expressing full potential, (3) unity with others, and (4) serving others. *Developing and becoming self* is about being true to oneself and becoming one’s higher self or a better person, and thus comes close to the definition of psychological need for autonomy. *Expressing full potential* is about being able to express one’s talents and creativity through one’s work and having a sense of achievement at work, and thus is conceptually close to the psychological need for competence. *Unity with others* is about being able to work together with others and about work organization as community, and thus is conceptually close to the psychological need for relatedness. Finally, *serving others* is about making a difference through one’s work and serving the needs of humanity, and thus comes close to what we call here beneficence. However, while Lips-Wiersma and Wright (2012, p. 659) argue that these dimensions “make up MFW [meaningful work] itself,” wee see these as psychological satisfactions that serve as important *sources* of meaningful work. Nevertheless, the conceptual overlap with her four dimensions of meaningful work and the currently examined four psychological satisfactions means that our present empirical investigation can also be used to offer indirect empirical support for Lips-Wiersma’s theoretical framework, the dimensions of which have not been previously empirically tested as sources of meaningful work.

Lips-Wiersma and Wright (2012, p. 659) are not the only one’s proposing such a list of four conditions of meaningful work: in one of the most comprehensive review of the meaningful work literature up to date, Rosso et al. (2010, p. 113), came to identify four “main pathways through which meaningful work is created or maintained.” First of these, *self-connection*, is about authenticity, self-concordance, and being in close alignment with how one sees oneself. This is conceptually close to the definition of autonomy used here. *Individuation*, in turn, is about self-efficacy, competence, and being able to conduct actions that produce intended effects and “define and distinguish the self as valuable and worthy” (Rosso et al., 2010, p. 115). Although there is conceptual overlap between this definition of individuation and the need for competence, the latter seems to be slightly more narrow category in not including self-esteem and distinguishing self “as valuable and worthy” that Rosso et al. (2010, p. 115) also include within individuation. Along with Ryan and Brown (2003; see also Ryan and Deci, 2017), we see self-esteem as an indicator of need satisfaction rather than as a need in its own right, and thus do not include it into the psychological needs. *Unification* is about belongingness, interpersonal connectedness, social identification with others, and more generally about being in harmony with other beings or principles. Finally, *contribution* is about the perceived impact of one’s work, transcendence and doing work in the “service of something greater than the self” (Rosso et al., 2010, p. 115), which is conceptually close to our definition of beneficence. However, pace Rosso et al. (2010), we would not include sense of transcendence into this dimension, again because we see it more as a potential outcome of making a contribution than as a psychological need in its own right.

Although we noted certain key differences in the definitions given by Rosso et al. (2010) for their four dimensions or pathways for meaningful work, and the way we defined autonomy, competence, relatedness, and beneficence in the current paper, our empirical investigation can also be used to offer indirect empirical support for this theoretical framework. More generally, these visible similarities between the four proposed pathways to meaning in life (Martela et al., 2017), and the four proposed pathways to meaningful work strengthens the case for suggesting that meaningfulness as regards life and meaningfulness as regards work are fundamentally the same psychological construct (Baumeister, 1991; Steger and Dik, 2009). Finally, it is worth noting that two of the satisfactions studied here – autonomy and competence – are highly similar to two dimensions of psychological empowerment at work, namely self-determination and competence (Spreitzer, 1995; Spreitzer et al., 1997). At the same time, psychological empowerment doesn’t have a dimension corresponding with relatedness. And although it has a dimension called ‘impact’ its definition deviates from how beneficence is defined here. For Spreitzer (1995, p. 1443–1444), impact is about the degree to which the individual ”can influence strategic, administrative, or operating outcomes at work,” and thus not about the positive contribution as such. Thus it might be interesting to study how closely related empowerment and meaningfulness are, and how the two additional satisfactions studied here, relatedness and beneficence, would relate to empowerment.

### Theoretical and Practical Contribution

Taken together, these results make important contributions to several research fields. First, as regards research on meaningful work, they provide empirical evidence for the importance of the four proposed satisfactions as key pathways to meaningful work. There have been calls for testing multiple potential predictors simultaneously (Rosso et al., 2010), but many interesting theoretical suggestions about potential sources have remained untested. Here we connect research on meaningful work with research on meaning in life and psychological wellness by showing that four much studied sources of well-being and meaning – the three psychological needs suggested by self-determination theory and beneficence as a sense of prosocial impact – are also robustly connected to meaningful work. Naturally, because our results are based on cross-sectional data and are thus correlative, longitudinal studies are needed to further clarify the causality.

The present research contributes also to the cross-cultural understanding of meaningful work by examining the same four psychological factors in three different countries. Self-determination theory, and the cross-cultural research conducted within that tradition (e.g., Chirkov et al., 2003; Chen et al., 2015), posit that the three needs for autonomy, competence, and relatedness are universal. Similarly cross-cultural research on beneficence has come to suggest it as a universal source of well-being (Aknin et al., 2013, 2015). Accordingly, we predicted that the four satisfactions will play a similar role in all three countries. Except for competence in United States, this turned out to be true. The connection between these four satisfactions and meaningful work thus does not seem to be confined to only one country, as it seems to be visible in at least these three countries. As regards why competence was not significantly related to meaningful work in United States, when controlling for the three other satisfactions, we can only speculate. Interestingly, previous research in United States has shown that competence has an independent predictive role in explaining people’s meaning in life evaluations (Martela et al., 2017), so the non-significant relation between competence and meaningfulness might be something specific to work. The zero-order correlation between meaningful work and competence was 0.49 so it could be the case that especially the strong relation between autonomy and meaningful work (standardized coefficient 0.56) did not leave room for competence to have any effect on meaningful work because of the intercorrelations between autonomy, competence, relatedness, and beneficence. However, as a *post hoc* control, we tested for an interaction effect between autonomy and competence, but didn’t find any. Another possibility is that people who are competent are more frequently externally rewarded with status and higher salary. In a materialistic culture like United States these factors might make people stay even in work that they do not find particularly meaningful. This is of course is only speculation and calls for future research on the topic. In any case, this result highlights the importance of conducting cross-cultural research. Participants in United States as compared to participants in Finland and India seem to draw on slightly different psychological factors when evaluating work meaningfulness.

The fact that the results from Finland and India were similar is especially interesting given that in the usual classifications of different cultures, Finland and United States are placed in the same Western or Protestant cluster, while India is seen as culturally more distant from these two (e.g., Tay and Diener, 2011). The similarity of results between Finland and India thus support the cross-cultural validity of the four satisfactions as predictors of meaningful work. Finally, as regards cross-cultural differences, it is interesting to note that in the United States the standardized coefficient for autonomy (0.56) was the highest, while the standardized coefficients for other satisfactions were smaller (0.22 or less). In contrast, in Finland the standardized coefficient for beneficence (0.46) was the highest, and the standardized coefficients for the other three satisfactions were smaller in size (between 0.15 and 0.18). In India, the standardized coefficients for different satisfactions were more in balance (between 0.13 and 0.29). Although the significance of these differences is hard to quantify, they seem to suggest that when thinking about meaningfulness of work, people in United States tend to emphasize sense of autonomy, people in Finland tend to emphasize sense of contribution, while people in India draw more equally from all four satisfactions. This is something that merits to be investigated further in future research and once again, qualitative research could provide some insights not easily captured through survey research.

Beyond research on meaningful work and cross-cultural research on the psychological experience of work, the present work also contributes to the research on basic psychological needs in organizations. The basic psychological needs, and self-determination theory more generally, have been investigated in work context in multiple studies (reviewed in Van den Broeck et al., 2016; Deci et al., 2017), but we extend this research by looking at a new outcome variable, meaningful work, and by examining beneficence as a fourth type of psychological satisfaction along with the three basic needs. At the same time the present work also extends research on the importance of prosocial behavior for the well-being of the employees (Bolino and Grant, 2016) by empirically examining the connection between a sense of prosocial impact and meaningful work. Finally, by using a novel dependent variable – meaningful work – this research also complements recent psychological investigations that have looked at whether beneficence is connected to subjective well-being and vitality (Martela and Ryan, 2016b), and meaning in life (Martela et al., 2017), when looked alongside with autonomy, competence, and relatedness.

Beyond the theoretical contributions, advancing understanding of the factors contributing to meaningful work is also important from a practical point of view. This is especially true given the changes in working life outlined in the introduction. Having a better understanding of the key factors that make work meaningful makes the goal of building organizations and policies supportive of meaningfulness more attainable. Meaningfulness as such can seem ‘abstract’ and hard to put into practice, but building practices and structures to support autonomy, competence, relatedness, and beneficence is already a more concrete goal. In supporting these satisfactions, one can take advantage of the existing literature on how to strengthen these factors in organizations (Deci and Flaste, 1995; Pink, 2010; Deci et al., 2017). Thus, the idea of four satisfactions underlying our sense of meaningfulness at work holds practical promise in giving managers, policy-makers, and other practitioners more executable suggestions on how to support employees’ sense of meaningfulness. This is also a hypothesis that can be tested empirically.

### Limitations

In interpreting the present results a few limitations must be acknowledged. First, the studies were based on self-reports, which might invite some common-method bias. Although we are not aware of any objective measures of the psychological satisfactions or meaningful work, it would be beneficial to try to overcome this reliance on self-reports in the future. Second, although our study included participants from three different countries from three different continents, this is still a narrow representation of the whole human population. Furthermore, all participants were from industrialized societies and/or had access to a computer and Internet. To further broaden our insight about what work means for people and how people make sense of meaningfulness of work, investigations into non-WEIRD populations (Henrich et al., 2010) would be illuminating. Also, Studies 1 and 2 used a different scale to assess meaningful work. While this usage of multiple scales adds to the robustness of the results in general, it makes the comparison between the results of Studies 1 and 2 harder. Fourth, the scales used asked about people’s sense of meaningfulness, but it is an open question how similarly or dissimilarly people interpret the constructs ‘meaningfulness’ and ‘meaningful work’ in different cultures. Thus it would be important that future work would examine more directly how these constructs are understood in different cultures and in different languages. Finally, given the proposed role of the four satisfactions as psychological pathways to meaningful work, it would be interesting to investigate the extent to which these four factors can mediate the relations between previously established organizational sources of meaningful work (e.g., Schnell et al., 2013) and the experience of meaningful work itself.

## Conclusion

What makes work meaningful is a key question in a time where work has become a key source of meaningfulness (Baum and Stewart, 1990; Baumeister, 1991; Steger and Dik, 2009), but where automatization and other developments threaten to significantly change how people work and whether there is work left for them to do (e.g., Brynjolfsson and McAfee, 2014; Frey and Osborne, 2017). Accordingly, the present article has suggested and empirically tested the proposition that four psychological factors – autonomy, competence, relatedness, and beneficence – would substantially determine how much meaningfulness people derive from work. The results from three different countries, Finland, India, and United States, by and large support the proposal. These results underscore what certain philosophical thinkers have already emphasized a long time ago. In looking for meaningfulness, we look for the intrinsic qualities of life that go beyond mere survival (Camus, 1955; Tolstoy, 2000). Meaningfulness, at the end of the day, is about finding intrinsic reasons to live. In this sense, self-expression through autonomy and competence as well as connecting to other people through caring relationships and through being able to contribute to the society indeed seem like prime candidates for what makes life worth living. In this sense, we hope to have given some empirical reasons to believe that such philosophical insights indeed might have a grain of truth in them.

## Author Contributions

FM and TR designed the studies together and finalized the analyses and the paper. TR collected the data for Study 1. FM collected the data for Study 2, did the initial analysis, and wrote the first draft of the paper.

## Conflict of Interest Statement

The authors declare that the research was conducted in the absence of any commercial or financial relationships that could be construed as a potential conflict of interest.

## Footnotes

1 Sometimes one also examines the χ^2^/df ratio, where <2 is taken as an indication of good fit and <3 an indication of acceptable fit (Schermelleh-Engel et al., 2003). Using this standard both the presently examined model and most other models examined in this article fail to reach adequate fit. However, Schermelleh-Engel et al. (2003, p. 33) themselves note that χ^2^ values increase with sample size and thus the “problem of sample size dependency cannot be eliminated by this procedure.” Accordingly, this method might be too conservative for large sample sizes.

2 To examine the sensitivity of the results to the chosen analysis method, based on a suggestion of one of the reviewers, we did a *post hoc* analysis using structural equation modeling where all four satisfactions were set to correlate with each other and set to predict meaningful work. The model fit was acceptable [χ^2^ (df = 142) = 645.97, CFI = 0.929, TLI = 0.915, RMSEA = 0.077, SRMR = 0.053] with autonomy (β = 0.447, *p* < 0.001), competence (β = 0.116, *p* = 0.006), and beneficence (β = 0.318, *p* < 0.001) significantly, and relatedness marginally significantly (β = 0.067, *p* = 0.085), predicting meaningful work. Thus these results mainly supported the same conclusions as the regression analysis.
